# Factors associated with retention of community health workers in maternal, newborn and child health programme in Simiyu Region, Tanzania

**DOI:** 10.4102/phcfm.v10i1.1506

**Published:** 2018-08-02

**Authors:** David P. Ngilangwa, George S. Mgomella

**Affiliations:** 1 Amref Health Africa, Dar es Salaam, Tanzania; 2 Department of Public Health and Primary Care, University of Cambridge, United Kingdom

## Abstract

**Background:**

Attrition of community health workers (CHWs) continues to threaten the full realisation and sustainability of community-based health programmes globally.

**Aim:**

This study aimed to understand factors associated with CHWs’ recruitment and their retention.

**Setting:**

This study was conducted in five districts of the Simiyu Region, namely, Bariadi, Busega, Itilima, Maswa and Meatu in north-western Tanzania.

**Methods:**

In this cross-sectional study design, 341 CHWs who were working with the maternal health programme were randomly selected. Semi-structured questionnaires were administered to all participants. Data were descriptively and inferentially analysed using SPSS version 20.

**Results:**

Majority (58.0%) of CHWs were below 35 years. Over half (53.1%) had completed primary education only. Motivation factors for being CHW were aspiration to serve the community and desire for further training to become a qualified medical practitioner. Community recognition and financial incentives were among the key retention reasons for the CHWs. Being married (odds ratio [OR] 5.9, 95% confidence interval [CI] 1.7–20.1) having prior volunteer experience (OR 10.5 95% CI 12.7–40.5) and prior employment OR 21.8 (CI 12.2–38.9) were positively associated with retention of CHWs, while being a female was negatively associated with retention OR 0.4 (CI 0.2–0.8).

**Conclusions:**

Both financial and non-financial incentives were critical in contributing to the retention of CHWs. Thus, health programmes should carefully select CHWs by understanding their motives beforehand, and provide them with incentives.

## Introduction

The dearth of human resources for health (HRH) coupled with high maternal and child mortality in low-and middle-income countries has necessitated an increased interest in the use of community health workers (CHWs) to improve health outcomes.^1^ A CHW is a member of the community who has not been formally trained as a clinician and has been chosen by his or her community to promote or provide health care services in the same community following a structured short-term period of training.^2^ During the Alma Ata Conference in 1978, CHWs were identified as one of the cornerstones of comprehensive primary health care (PHC).^3^ Over the past three decades, a number of studies have shown that CHWs can help to reduce morbidity and mortality related to HIV and AIDS, malaria and tuberculosis.^3,4,5,6,7,8,9,10,11,12,13^ More studies across sub-Saharan Africa documented CHWs’ effectiveness in diagnosis of malaria and accurate assessment of ailments among neonates for immediate referral to health care services.^12,14,15,16^

Studies from Kenya, Nigeria, Bangladesh, India and Indonesia have showed that CHWs contributed to the reduction of maternal, neonatal and child mortality through antenatal care uptake, anti-tetanus vaccination coverage during pregnancy and increasing deliveries within health facilities. Community health workers also increased the number of women breastfeeding within the first 24 h, number of newborns checked by health workers within 48 h and increased the knowledge of specific newborn danger signs among mothers.^15,17,18,19,20^ Because of these successes, many countries in sub-Saharan Africa and Southeast Asia are planning, implementing and scaling up CHW programmes at the national level.

However, CHW programmes face challenges, namely low attraction and motivation for new CHWs, including poor training, inadequate supervision, lack of supplies and poor relationships with communities.^3,21,22,23^ One of the most frustrating elements of many CHW programmes is their high attrition rates.^3^ Globally, the reported annual CHW attrition rates are between 3.2% and 77.0%, and it is mainly thought to be because of lack of appropriate remuneration, as CHWs are expected to be volunteering their time.^2,3^ Such high attrition rates lead to lack of continuity in the relationships established among CHWs, community and health systems; decreased stability of the programmes; increased costs of CHW replacements in relation to identifying, screening, selecting and training new CHWs; and lost opportunities to build on experience.^24^ Indeed, the effectiveness of CHWs significantly depends on retention.^3^ Retention of CHWs is critical for health system performance; however, the key issue is how best the volunteer-based programmes can attract, motivate and retain their CHWs.^25^ There is a growing curiosity to explore the links between incentives, motivation and retention of CHWs in developing countries.^2^ This, in turn, leads to a call for a better understanding of what motivates individuals to become and remain a CHW.^26^

While Tanzania has the highest density of PHC facilities in Africa, equitable access and quality of care remain a challenge.^27^ Tanzania has been a pioneer in establishing community-level health care services countrywide, yet challenges remain in sustaining these systems and ensuring adequate human resource strategies.^28^ Thus, the Tanzanian government has emphasised the importance of community involvement to improve PHC coverage. To help achieve this coverage, CHW programmes have been implemented mainly by non-governmental organisations (NGOs) assisting the government since the 1970s, resulting in a cadre of CHWs with members that differ in length of service, training, capabilities and sources of support.^26^

In addition, CHWs in Tanzania are still working as volunteers and are not considered as public servants in the health care system. However, in the past few years, there have been efforts in the country which have led to the recent formalisation of the CHW cadre, including the approval of the community-based health programme (CBHP) policy in 2014. The policy will standardise the fragmented CHWs’ activities across the country.^28^ Thus, the interest in understanding determinants of CHWs’ attraction, motivation, retention and attrition in Tanzania to inform policy and programmes has been given a strong impetus.^26^

## Description of Amref Health Africa’s ***Uzazi Uzima*** Programme

For the 4 years (2011–2014), Amref Health Africa has been supporting the Tanzanian government targets to strengthen the delivery of health services to women and children in five districts of the Simiyu Region, north-western Tanzania. To achieve the expected results in this region, Amref Health Africa worked with local governments and partners to support Tanzania’s health systems by improving the quality and increasing the access to quality Maternal Neonatal and Child Health (MNCH) services by strengthening formal and community health systems through a programme known as *Uzazi Uzima* which in English means giving birth is giving life.

The programme targeted to reach 108 353 pregnant women, 21 010 neonates and 114 575 children aged below 5 years as direct beneficiaries. To reach all beneficiaries, the *Uzazi Uzima* programme recruited and trained 3924 CHWs – 2000 men and 1924 women – thus exceeding the national target (i.e. having two CHWs per village). Each CHW was expected to serve 60–120 households, depending on the size of the village.^28^ All CHWs were trained for 1 week using the curriculum developed by the Ministry of Health and Social Welfare. The curriculum covered topics on antenatal and postnatal counselling, early detection of pregnancy, danger signs during pregnancy and facility delivery and postnatal care. The trained CHWs visited households with pregnant women or children below the age of 5 years to provide preventive health education, counselling and collection of data. Where relevant, CHWs facilitated referrals to health facilities. The time spent in visiting their respective households was 15 h a week which enabled them to visit 40 households on average.

The programme offered CHWs working tools like gumboots, torches, umbrellas, bags and notebooks. All CHWs were attached to the respective nearby health facilities. The health facility managers, in most cases nurse-midwives or clinical officers, were the immediate supervisors of CHWs’ daily community works. The supervisors provided mentorship and other technical support as needed on areas related to Integrated Management Childhood Illness (IMCI); MNCH; sensitisation and mobilisation; and community-based health management information systems (CBHMIs). Monthly data collected were submitted to the health facilities and then aggregated. Aggregated data were then escalated to district and regional levels. In addition, CHWs met with the programme staff on a monthly basis for submission of reports and supportive supervision. They received a total of TZS 10 000 (approximately US$5) as reimbursement for transport and lunch. However, by the third year of implementation, 500 CHWs were reported to be inactive or had been not working for a long time within the programme.

In this article, we report on the factors that facilitate CHWs to remain active and the magnitude of attrition throughout the implementation of the MNCH programme in the Simiyu Region in Tanzania. The generated evidence will be compared with that found elsewhere to increase the body of knowledge in the subject matter.

## Methods

### Study design and settings

**FIGURE 1:**
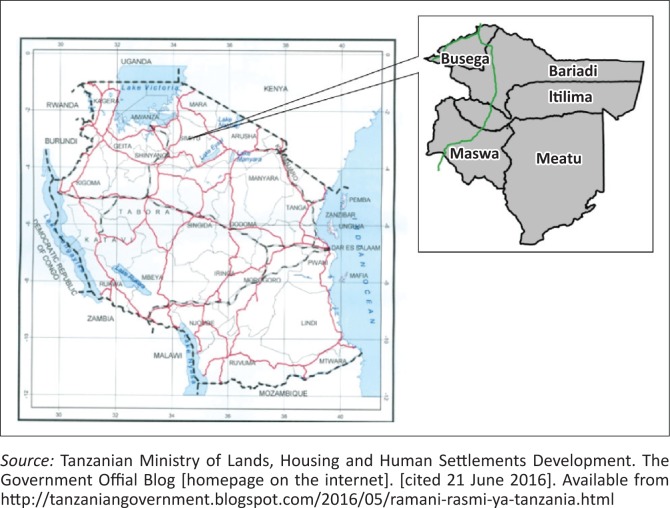
Map of Tanzania showing the administrative districts of the Simiyu Region.

A cross-sectional study to determine the magnitude and factors associated with CHWs’ retention was conducted in the Simiyu Region in north-western Tanzania from September to November 2014. The region is administratively divided into five districts, namely Bariadi, Maswa, Itilima, Meatu and Busega, with Bariadi Town being the regional administrative headquarters (Figure 1).^29^ The five districts have 111 wards and 479 villages, and serve with 224 health facilities, both private and public. The Simiyu Region has a population of 1 584 157 people – 824 266 women and 759 891 men.^30^ Subsistence farming and pastoralism are the main sources of livelihood in the region.

### Study population

All active CHWs recruited and trained by Amref Health Africa’s *Uzazi Uzima* programme in the Simiyu Region were included in this study. In order to meet the objectives of our study, we recruited CHWs who had completed a week’s basic training course on MNCH and who were registered and had served as CHWs for the duration of at least 6 months and freely consented to participate in the study.

### Sampling

We used simple random sampling method to select study participants from the *Uzazi Uzima* programme’s records. Any CHW whose name appeared in the programme’s incentive recipients list was regarded to be an active CHW, as they were reporting and regularly meeting their respective supervisors. Community health workers that were not in the list or did not report to their respective supervisors for the past 3 months were considered to have dropped out (attrition).

The study used a multistage random sampling method to obtain respondents from each of the five districts of the Simiyu Region. The sample frame for active CHWs was the list of those recruited and currently working as indicated by the presence in the list of CHWs receiving incentives and actively reporting to their respective health facilities for the period of 6 months prior to the study.

The sample size was calculated using OpenEpi, A Web-based Epidemiologic and Statistical Calculator for Public Health.^31^

*n* = [DEFF × Np(1-p)] / [(d^2^ / Z^2^_1-α/2_ × (N-1) + p × (1-p)]

where:

*n* = sample size

D = design effect (apply the value of 1)

Z = confidence level 95% (standard value = 1.96)

p = prevalence of retention in CHWs programmes = 90%

d = desired precision of 5% (0.05).

Sample size was calculated in two stages: firstly, sample size of all active CHWs for the whole region of Simiyu was calculated at 267. Taking into consideration the estimated non-response rate of 10%, we adjusted the sample size to 294. Secondly, we calculated the proportion for each district by dividing the total number of active CHWs in a district with the number of active CHWs in the district multiplied by sample size for 3424 CHWs. For example, for Bariadi district, the calculation is as follows: 809 / 3424 × 267 = 63 (see Table 1).

**TABLE 1:** Distribution of sampled community health workers in the Simiyu Region, 2014.

District	Number of active community health workers	Number of sampled community health workers	Community health workers interviewed
Bariadi	809	63	97
Busega	390	30	37
Itilima	735	57	73
Maswa	730	58	77
Meatu	760	59	57

**Total**	**3424**	**267**	**341**

## Data collection

In order to ensure the validity and reliability of the data collection tool, the semi-structured questionnaire was developed by the study team mainly by adapting questions from studies in Bangladesh.^24,32^ Questions on socio-demographic variables, recruitment strategies and factors that supported CHWs to continue working were selected. The content and construct of the semi-structured questionnaire was validated by an expert panel that consisted of epidemiologists and social scientists. The semi-structured questionnaire was initially prepared in English and then translated to Kiswahili (the Tanzanian national language which is widely spoken). Later, the semi-structured questionnaire was back translated to English by an independent English native speaker. The semi-structured questionnaire was then piloted in Bariadi district by administering it to 20 CHWs, by ensuring that those participants were not included in the study. Thereafter, issues from the pilot were taken on board to improve the questionnaire by rewording and omitting some sections.

Trained research assistants used pretested, semi-structured questionnaires to collect quantitative data from sampled CHWs. Data collected were socio-demographic information– age, area of residence, level of education, marital status and employment; supporting mechanisms included training, supervision and supplies. Others were financial and non-financial incentives – working hours, incentives, support from the organisation, respect from the community, social recognition, benefits, leave, pay, changes in social prestige and family and community approval. In addition, the *Uzazi Uzima* programme data were used to calculate magnitude of attrition and retention proportions.

In this study, data validity and reliability were ensured by the field supervisor and principal investigator who reviewed all the questionnaires on a daily basis to rectify any inconsistencies and ensure that intended questions have been answered properly. Feedbacks emanating from review of questionnaire interviews were conveyed to the research assistants for the improvement of quality of data collected.

### Data management and analysis

Data were double entered using EPI Info (CDC, Atlanta, GA, USA) screens and stored in MS Access database (Microsoft Corporation, WA, USA). The data were classified and organised according to district, ward and nature of residences – semi-urban or rural in order to facilitate comparison with other demographic characteristics. We also checked for data completeness and consistency in order to detect and correct any missing information.

Data were cleaned, edited and analysed by SPSS 20 (IBM Corporation, NY, USA). We performed descriptive analysis and presented means, medians, ranges, standard deviations and proportions. Frequency tables and cross-tabulation were produced in order to describe the demographic characteristics and other factors associated with retention in the programme among the CHWs in the Simiyu Region. Attrition rates were obtained by calculating the ratio between the total number of CHWs who dropped out of the programme as a numerator and those who were recruited and trained in the 4 years of the implementation as a denominator.

Moreover, the study compared proportions of retention of CHWs across the five districts of the Simiyu Region, their level of education and exposure to other opportunities. Bivariate logistic regression analysis was conducted to test associations between retention and socio-demographic characteristics with odds ratio with 95% confidence intervals. Chi square was used to test observed statistical differences among the groups. A *p*-value of less than 0.05 was regarded as statistically significant.

### Ethical considerations

Ethical clearance to conduct this study was obtained from the Amref Health Africa Institutional Review Board (IRB/AMREF/2014/04/01) and permission to conduct the study was obtained from local government authorities in the Simiyu Region. Written informed consent was obtained from research participants. The study also obtained permission from the local government authorities in the respective districts before data collection.

## Results

### Socio-demographic characteristics of the study participants

In total, 341 of the active CHWs sampled agreed to participate in the study; this includes an additional 47 to those CHWs initially sampled for interviews. The mean age of the CHWs recruited in Simiyu was (33 ± 9.57) years, the youngest being 18 years and the oldest 63 years. Among the active CHWs, there were 184 females (54.0%).

Generally, about half (164,48.1%) of the active CHWs had served the *Uzazi Uzima* programme for at least 1 year. With regard to education level, 160 (46.9%) of them had attained a secondary level of education with over half of the CHWs having attained only a primary level of education. The main source of their livelihood was subsistence farming as reported by 231 (67.7%) of the CHWs. Almost all CHWs were born in the villages they had been serving. Socio-demographic characteristics of the participants are shown in Table 2.

**TABLE 2:** Socio-demographic characteristics of community health workers working with the *Uzazi Uzima* programme in the Simiyu Region, Tanzania.

Characteristics	*n*	%
**Age (years)**		
≤ 35	198	58.0
≥ 36	143	42.0
**Sex**		
Male	46	157
Female	54	184
**Education (years)**		
Primary(7)	181	53.1
Secondary(11)	160	46.9
**Ethnic group**		
Sukuma	323	94.7
Others	17	5.3
**Distribution of community health workers by district**		
Bariadi	97	28.4
Maswa	77	22.6
Itilima	73	21.4
Meatu	57	16.7
Busega	37	10.9
**Marital status**		
Married	255	74.8
Widowed	12	3.5
Single	61	17.9
Others	12	3.8
**Have children and/or dependants**		
Yes	297	87.1
No	44	12.9
**Number of children and/or dependants**		
≤ 3	97	28.4
≥ 4	200	71.6
**Sources of earning**		
Subsistence farming	231	67.7
Petty trade	60	17.5
Others	50	14.6
**Monthly household income in Tanzanian Shilling (TZS) (US$)**		
≤ 150 000	206	60.4
≥ 150 001	29	8.5
Not disclosed	106	31.1
**Years of experience as community health workers**		
≤ 1 year	175	51.9
≥ year	164	48.1

### Attrition rate

**FIGURE 2:**
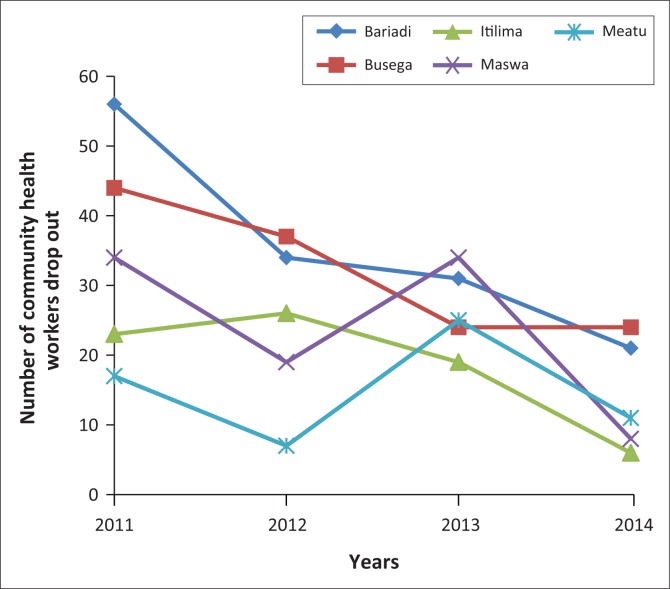
Trend of community health worker attrition in the Simiyu Region from 2011 to 2014.

*Uzazi Uzima* programme’s data recorded an attrition rate of 12.7% (500/3924) over 4 years among CHWs who were recruited. Our programme data showed that in the first year 174 CHWs left, while 123 and 133 CHWs left in the second and third year, respectively. The highest dropout was 20.1% (129/616) observed in Busega district followed by 17.0% (142/833) and 11.6% (95/820) as reported from Bariadi and Maswa districts, respectively. Among the reasons given for such attrition among the CHWs (about 70 of them) who were interviewed by the programme staff, the reasons mentioned for termination of their service were relocation to other places because of marriage or agricultural activities and inadequate time to do the voluntary job. Figure 2 depicts the trend on attrition over 4 years.

### Sources of recruitment information

We investigated how CHWs heard about the programme. The majority of active CHWs reported that they saw an advertisement on the village notice board, followed by those who got information through the village officials (*p* = 0.029). In addition 188 (55.1%) CHWs reported that their aspiration to serve the community was the key motivation factor for them to join the *Uzazi Uzima* programme, followed by the desire for further training to be a qualified medical practitioner 82 (24%) and desire to acquire further knowledge on MNCH 39 (11.4%). Other motivational factors were desire to use available time productively and desire for financial gain (*p* = 0.005). Table 3 presents findings from 341 CHWs.

**TABLE 3:** Recruitment and factors that influenced an individual to become a community health worker. *N* = 341.

Variables	*n*	*%*	*p*
**Where and how did you get information regarding the recruitment of this position?**			
Advertisement at village notice board	177	51.9	0.029
Through village executive officials	158	46.2	
Through the *Uzazi Uzima* programme staff	5	1.4	
Others	1	0.2	
**What motivated you to work with the *Uzazi Uzima* programme as a community health worker?**			
To serve the community	188	55.1	0.005
Desire for further training to be qualified medical practitioner	82	24.0	
Desire to acquire further knowledge on maternal, neonatal and child health	39	11.4	
Desire to use available extra time for the community	10	2.9	
To be recognised by community members	7	2.0	
My family members and friends encouraged me to apply	6	1.7	
Desire to acquire financial gain	4	1.1	
Was unemployed/had extra time	2	0.5	
**What was your reaction towards roles of community health workers when you joined the programme?**			
Manageable	309	90.8	< 0.001
Not manageable	28	8.2	
Was not sure	3	0.8	
**Have you ever been employed by any organisation before joining the *Uzazi Uzima* programme as community health worker?**			
Yes	273	80.0	0.073
No	68	20.0	
**If yes, employment was on what basis?**			
Paid	19	80.2	0.098
Volunteerism	77	19.8	

The study also analysed the factors that motivated one to continue serving as a CHW for the entire duration of the project. We found that the majority of them cited that the programme had met their desires of joining (191, 56.0%), receiving the necessary support from the community and programme (251, 73.8%) and recognition from the community, (156, 45.7%), as shown in Table 4.

**TABLE 4:** Factors for retention among community health workers working for the *Uzazi Uzima* programme in the Simiyu Region, Tanzania. *N* = 341.

Variables	*n*	%	*p*
**How many hours are/were you spending as community health worker per week?**			
2–8 h	117	34.3	-
9–16 h	108	31.7	-
17–20 h	61	17.9	-
> 21	55	16.1	-
**Total**	**341**	**100.0**	< 0.001
**Did the project/work of community health worker meet your desires of joining it?**			
Yes	191	56.0	-
No	134	39.3	-
Not sure	16	4.7	-
**Total**	**341**	**100.0**	< 0.001
**In performing your daily roles, are/were you receiving any support from village or the project?**			
Yes	251	73.8	-
No	89	26.2	-
**Total**	**340**	**100.0**	< 0.001
**What are the reasons that prompt you to continue being a community health worker?**			
Recognition from the community	156	45.7	-
Financial incentive	89	26.1	-
Enjoying the work	51	15.0	-
Expectation of getting a better job from experience gained as community health worker	20	5.9	-
No opportunities for further studies	12	3.5	-
Others (support from family, supervisors, work not too demanding)	13	3.8	-
**Total**	**341**	**100.0**	< 0.001

In Table 5, we carried out a logistic regression to predict CHWs who will remain working in the programme. In general, being married (OR 5.9, 95% CI = 1.7–20.1) and prior volunteer experience (OR 10.5 95% CI = 12.7–40.5) were associated with being retained as a CHW. In contrast, married women were less likely when compared to the men to remain as CHWs.

**TABLE 5:** Predictors of community health workers to continue working with the *Uzazi Uzima* programme.

Variable	*N*	*%*	Odds ratio	*p*-value
**Sex**				
Male	161	45.6	1.0	< 0.001*
Female	192	55.4	0.4 (0.2–0.8)*	-
**Marital status**				
Currently single	94	26.6	1.0	0.004*
Married	259	74.4	5.9 (1.7–20.1)*	-
**Education**				
Primary	184	52.1	0.9 (0.3–12.7)	0.070
Secondary	169	47.9	1.0	-
**Prior employment**				
Yes	285	80.7	1.0	< 0.001*
No	69	19.3	21.8 (12.2–38.9)*	-
**Prior volunteerism experience**				
Yes	79	22.4	1.0	< 0.001*
No	274	77.6	10.5 (12.7–40.5)*	-
**District of residence**				
Bariadi	101	28.6	1.0	0.160
Others districts	252	71.2	0.7 (0.4–1.1)	-

* , statistically significant values.

### Recommendations for retention of community health workers

We inquired from active CHWs about their recommendations for the programmes using CHWs to reduce attrition and meet the targets. Provision or increment of monetary incentive was ranked as the number one recommendation; it was mentioned by 200 (61.7%) CHWs, while provision of regular refresher training and convening meetings with villagers to educate them about CHWs and their importance to the community were ranked number two and three, respectively. In addition, timely provision of working tools and regular supportive supervision were also important. This finding was statistically significant (< 0.001) (Table 6).

**TABLE 6:** Recommendations for retention of community health workers working with the *Uzazi Uzima* programme in the Simiyu Region, Tanzania.*N* = 324.

Variable	*n*	%	*p*-value
Increase incentives	200	61.7	< 0.001
Provide regular and refresher trainings	49	15.1	-
Arrange meeting with villagers to educate on community health workers	40	12.3	-
Provide adequate and timely support in supplies and other necessities	19	5.9	-
Provide regular supportive supervision	7	2.2	-
Arrangement transfer for those who relocate within project catchment	6	1.9	-
Others	3	0.9	-

*N*, number of community health workers.

## Discussion

In this study, we found the attrition rate among recruited CHWs, over 4 years of the implementation of the *Uzazi Uzima* programme, to be 12.7%. It is evident that the attrition rate in our study was within the range reported for similar programmes implemented elsewhere which was between 3.2% and 77%.^3^ Moreover, other programmes in Bangladesh, Ghana and Kenya reported dropout rates ranging from 21% to 41%, which is higher than that found in our study. Among the reasons for such high rate of attrition was poor financial incentives to CHWs,^24,32,33,34,35^ and this reason was in line with our findings. Other main causes of attrition were interference with personal work, family pressure and a high workload.^24,31,34^ Our programme data showed that relocation to other places for employment or studies was an important cause of attrition among CHWs. The observed differences could be because of a number of reasons, including study designs, settings and differences in education levels among the CHWs who participated in the studies. As our study did not collect data from the inactive CHWs, we could not establish other factors that led to CHWs dropping out from the programme. Therefore, for the CHWs programme to combat high attrition rates, there is a need to regularly review financial incentives based on the availability of funds.

A rigorous process of selecting CHWs is among the key factors that determine retention within the programme.^31^ However, another study in Kenya experienced a high rate of attrition attributed to poor selection process of CHWs by village leaders.^34^ Thus, careful and participatory selection of CHWs is imperative if we want to sustain the CHW programmes. A study in Mexico found that when one is invited or asked to be a CHW, it is likely that he or she remains a CHW for the entire duration of the programme.^35^ The selection team should also be aware of CHWs’ ambitions and dreams during recruitment stages which should be paramount for the programme.

The majority of active CHWs in this study learnt about this opportunity through advertisements posted at the village government offices’ notice boards and the others got information through word-of-mouth from village government officials. This not only implies the ownership of the programme by the villagers and CHWs but also brings a sense of accountability to the community. Thus, a CHW felt obliged to serve the community. In addition, some CHWs cited that they continue to work as they are recognised by their community and feel accountable to the village.

In addition, findings from this study showed that a significant proportion of CHWs joined this cadre as a stepping stone towards their career goal in health care. They cited that their desire for further training to be a qualified medical practitioner was among the motivating factors to join the programme and continue working as CHWs. The Tanzanian Ministry of Health and Social Welfare recently approved the National Community Based Health Program Policy. The policy recognises this cadre and plans are underway to standardise training duration and entry qualifications across the country. However, there is a need to create clear career pathways for those who served as CHWs in a particular period of time. CHW programmes should either design or support bridging courses that will enable participants to upgrade their skills and education levels as a preparatory phase towards taking more advanced positions or training in the health care system in which they qualify.

Our findings showed that women were less likely to remain CHWs compared to men. This could be because the majority of our CHWs were below 35 years, with most of them being unmarried. Thus, when they get married probably their partners did not allow them to continue with the work. However, further studies should also plan to investigate this in order to find the exact reasons. In addition, we found that married CHWs were more likely to be retained than those who were single. This finding is consistent with research conducted by Alam et al. in the slums of Dhaka, Bangladesh.^24^ However, the main reasons could be that married CHWs are more stable and have established their families and even their businesses; thus, they are less likely to relocate to other places compared with their single counterparts.

Interestingly, we established that CHWs without prior work and volunteer experience were more likely to serve the community for longer periods compared with those with experience. On the contrary, we would have thought that individuals without volunteer and work experience would serve for a shorter period of time and leave to look for greener pastures; however, our study suggests the contrary. The main reasons for this could be either such CHWs have settled and are earning enough for their living or they have little exposure to the availability of other opportunities. We recommend further studies to ascertain our findings.

We did not find any association between education and retention. This is contrary to our notion that CHWs with primary education are more likely to stay in programmes, as they have limited opportunities related to education and career compared with their counterparts with secondary education. This calls for further research.

### Limitations of the study

In our initial design of this study, we intended to interview dropout CHWs to establish the determinants for the attrition. However, during the implementation, we faced challenges related to the high rate of non-responses from the dropout CHWs. The process of contacting and reaching out to dropout CHWs was challenging, as the majority had relocated out of the Simiyu Region. We made different attempts to reach them including traveling to their new places and even having phone interviews without success. Therefore, our findings were mainly confined to retention rather than attrition.

## Conclusion

Our study found that both financial and non-financial incentives, such as regular refresher training, supportive supervision, recognition of the communities they serve and bicycles were critical in contributing to the retention of CHWs. We recommend that CHW programmes carefully select CHWs and provide continuous and timely material incentives and regular remunerations to enable retention; where necessary, incentives have to be reviewed regularly.

The majority of the CHWs in this study felt that recognition from the community they serve is important, while others felt that experience gained from their work will be an added advantage in building their professional career. In this regard, it is critical for the Ministry of Health and other stakeholders to recognise the time one served in the community.

But again, imparting CHWs with entrepreneurship skills to run small and medium businesses in order to sustain their living is critical in order to retain them in service provision.

## References

[1] Global Health Workforce Alliance and World Health Organization Global experience of community health workers for delivery of health related millennium development goals: A systematic review, country case studies, and recommendations for integration into national health systems. 2010 Global Health Workforce Alliance and World Health Organization, Geneva, Switzerland.

[2] Nkonki L, Cliff J, Sanders D Lay health worker attrition: Important but often ignored. Bull World Health Organ. 2011;89(12):919–923. 22271950 10.2471/BLT.11.087825 PMC3260896

[3] Bhattacharyya K, Winch P, LeBan K, Tien M Community health worker incentives and disincentives: How they affect motivation, retention, and sustainability. Arlington, VA: Published by the Basic Support for Institutionalizing Child Survival Project (BASICS II) for the United States Agency for International Development;2001 10.2471/BLT.11.087825

[4] Yeboah-Antwi K, Pilingana P, Macleod WB, Semrau K, Siazeele K, Kalesha P, et al Community case management of fever due to malaria and pneumonia in children under five in Zambia: A cluster randomized controlled trial. PLoS Med. 2010;7(9):e1000340 10.1371/journal.pmed.1000340 20877714 PMC2943441

[5] Stromberg DG, Frederiksen J, Hruschka J, Tomedi A, Mwanthi M. A community health worker program for the prevention of malaria in eastern Kenya. Educ Health. 2011;24(2):474. 22081652

[6] Schneider H, Hlophe H, Van Rensburg D Community health workers and the response to HIV/AIDS in South Africa: Tensions and prospects. Health Pol Plann. 2008;23(3):179–187. 10.1093/heapol/czn006 18388133

[7] Mukherjee JS, Eustache FE Community health workers as a cornerstone for integrating HIV and primary healthcare. AIDS Care. 2007;19(Suppl 1):S73–S82. 10.1080/09540120601114485 17364390

[8] Ospina JE, Orcau A, Millet JP, Sanchez F, Casals M, Cayla JA Community health workers improve contact tracing among immigrants with tuberculosis in Barcelona. BMC Public Health. 2012;12:158 10.1186/1471-2458-12-158 22394990 PMC3312853

[9] Lefeuvre D, Dieng M, Lamara F, Raguin G, Michon C Community health workers in HIV/AIDS care. Sante Publique. 2014;26(6):879–888. 25629682

[10] Islam MA, Wakai S, Ishikawa N, Chowdhury AM, Vaughan JP Cost-effectiveness of community health workers in tuberculosis control in Bangladesh. Bull World Health Organ. 2002;80(6):445–450. 12132000 PMC2567545

[11] Kisia J, Nelima F, Otieno DO, Kiilu K, Emmanuel W, Sohani S, et al. Factors associated with utilization of community health workers in improving access to malaria treatment among children in Kenya. Malaria J. 2012;11:248 10.1186/1475 PMC3473249 22846194

[12] Bagonza J, Kibira SP, Rutebemberwa E Performance of community health workers managing malaria, pneumonia and diarrhoea under the community case management programme in central Uganda: A cross sectional study. Malaria J. 2014;13:367 10.1186/1475-2875-13-367 PMC4174662 25231247

[13] Mwai GW, Mburu G, Torpey K, Frost P, Ford N, Seeley J Role and outcomes of community health workers in HIV care in sub-Saharan Africa: A systematic review. J Int AIDS Soc. 2013;16:18586 10.7448/IAS.16.1.18586 24029015 PMC3772323

[14] Hawkes M, Katsuva JP, Masumbuko CK Use and limitations of malaria rapid diagnostic testing by community health workers in war-torn Democratic Republic of Congo. Malaria J. 2009;8:308 10.1186/1475-2875-8-308 PMC2804690 20028563

[15] Bari S, Mannan I, Rahman MA, Darmstadt GL, Serajil MH, Baqui AH, et al. Trends in use of referral hospital services for care of sick newborns in a community-based intervention in Tangail District, Bangladesh. J Health Popul Nutr. 2006;24(4):519–529. 17591349 PMC3001156

[16] Mubi M, Janson A, Warsame M, Martensson A, Kallander K, Petzold MG, et al. Malaria rapid testing by community health workers is effective and safe for targeting malaria treatment: Randomised cross-over trial in Tanzania. PLoS One. 2011;6(7):e19753 10.1371/journal.pone.0019753 21750697 PMC3130036

[17] Adam MB, Dillmann M, Chen MK, Mbugua S, Ndung’u J, Mumbi P, et al. Improving maternal and newborn health: Effectiveness of a community health worker program in rural Kenya. PLoS One. 2014;9(8):e104027 10.1371/journal.pone.0104027 25090111 PMC4121293

[18] Agrawal PK, Agrawal S, Ahmed S, Darmstadt GL, Williams EK, Rosen HE, et al. Effect of knowledge of community health workers on essential newborn health care: A study from rural India. Health Policy Plan. 2012;27(2):115–126. 10.1093/heapol/czr018 21385799 PMC3606030

[19] De Haas I, Moes N, Wolffers I Prevention of neonatal tetanus in developing countries hampered by local organization and limited knowledge of health personnel and traditional midwives; North Sulawesi (Indonesia). Ned Tijdschr Geneeskd. 1994;138(20):1032–1035. 8196802

[20] Findley SE, Uwemedimo OT, Doctor HV, Green C, Adamu F, Afenyadu GY Comparison of high- versus low-intensity community health worker intervention to promote newborn and child health in Northern Nigeria. Int J Womens Health. 2013;5:717–728. 24194649 10.2147/IJWH.S49785 PMC3814931

[21] Haines A, Sanders D, Lehmann U, Rowe AK, Lawn JE, Jan S, et al. Achieving child survival goals: Potential contribution of community health workers. Lancet. 2007;369(9579):2121–2131. 10.1016/S0140-6736(07)60325-0 17586307

[22] Mpembeni RN, Bhatnagar A, LeFevre A, Chitama D, Urassa DP, Kilewo C, et al. Motivation and satisfaction among community health workers in Morogoro Region, Tanzania: Nuanced needs and varied ambitions. Hum Resour Health. 2015;13:44 10.1186/s12960-015-0035-1 26044146 PMC4458000

[23] Nzioki JM, Onyango RO, Ombaka JH Efficiency and factors influencing efficiency of community health strategy in providing maternal and child health services in Mwingi District, Kenya: An expert opinion perspective. Pan Afr Med J. 2015;20:88 https://doi.org/10.11604/pamj.2015.20.88.4711 26090046 10.11604/pamj.2015.20.88.4711 PMC4450026

[24] Alam K, Oliveras E Retention of female volunteer community health workers in Dhaka urban slums: A prospective cohort study. Hum Resour Health. 2014;12:29 10.1186/1478-4491-12-29 24886046 PMC4040363

[25] Willis-Shattuck M, Bidwell P, Thomas S, Wyness L, Blaauw D, Ditlopo P Motivation and retention of health workers in developing countries: A systematic review. BMC Health Serv Res. 2008;8:247 10.1186/1472-6963-8-247 19055827 PMC2612662

[26] Greenspan JA, McMahon SA, Chebet JJ, Mpunga M, Urassa DP, Winch PJ Sources of community health worker motivation: A qualitative study in Morogoro Region, Tanzania. Hum Resour Health. 2013;11:52 10.1186/1478-4491-11-52 24112292 PMC3852396

[27] Ramsey K, Hingora A, Kante M, Jackson E, Exavery A, Pemba S, et al. The Tanzania Connect Project: A cluster-randomized trial of the child survival impact of adding paid community health workers to an existing facility-focused health system. BMC Health Serv Res. 2013;13(Suppl 2):S6 10.1186/1472-6963-13-S2-S6 PMC3668255 23819587

[28] Ministry of Health and Social Welfare National community based health program: Policy guidelines; towards a sustainable cadre of community health workers. Dar es Salaam, Tanzania: The United Republic of Tanzania;2014.

[29] Tanzanian Ministry of Lands, Housing and Human Settlements Development The Government Offial Blog [homepage on the internet]. [cited 21 June 2016]. Available from http://tanzaniangovernment.blogspot.com/2016/05/ramani-rasmi-ya-tanzania.html

[30] United Republic of Tanzania 2012 population and housing census: Population distribution by administrative areas. Dar es Salaam, Tanzania: National Bureau of Statistics and Office of Chief Government Statistician;2013.

[31] Dean AG, Sullivan KM, Soe MM OpenEpi: Open source epidemiologic statistics for public health [homepage on the Internet]. [Updated 2013 Apr 06;cited 2014 Feb 26]. Available from: www.OpenEpi.com

[32] Rahman SM, Ali NA, Jennings L, Seraji MH, Mannan I, Shah R, et al. Factors affecting recruitment and retention of community health workers in a newborn care intervention in Bangladesh. Hum Resour Health. 2010;8:12 10.1186/1478-4491-8-12 20438642 PMC2875202

[33] Abbey M, Bartholomew LK, Nonvignon J, Chinbuah MA, Pappoe M, Gyapong M, et al. Factors related to retention of community health workers in a trial on community-based management of fever in children under 5 years in the Dangme West District of Ghana. Int Health. 2014;6(2):99–105. 10.1093/inthealth/ihu007 24532651

[34] Olang’o CO, Nyamongo IK, Aagaard-Hansen J Staff attrition among community health workers in home-based care programmes for people living with HIV and AIDS in western Kenya. Health Policy. 2010;97(2–3):232–237. 20807685 10.1016/j.healthpol.2010.05.004

[35] Owek C, Abong’o B, Oyugi H, Oteku J, Kaseje D, Muruka C, Njuguna J Motivational factors that influence retention of community health workers in a Kenyan district. Publ Health Res. 2013;5(3):109–115.

[36] Ramirez-Valles J “I was not invited to be a [CHW]… I asked to be one”: Motives for community mobilization among women community health workers in Mexico. Health Educ Behav. 2001;28(2):150–165. 11265826 10.1177/109019810102800203

